# Endoscopic ultrasound-guided radiofrequency ablation for treatment of pancreatic neuroendocrine tumors: Multicenter prospective study

**DOI:** 10.1055/a-2689-5949

**Published:** 2025-10-16

**Authors:** Gianenrico Rizzatti, Bertrand Napoléon, Fabrice Caillol, Stefano Francesco Crinó, Germana de Nucci, Khanh Do-Cong Pham, Marc Giovannini, Sarah Leblanc, Silvia della Torre, Laurent Palazzo, Pia Clara Pafundi, Maria Cristina Conti Bellocchi, Cristiano Spada, Alberto Larghi

**Affiliations:** 1 18654 Digestive Endoscopy Unit, Fondazione Policlinico Universitario Agostino Gemelli IRCCS, Roma, Italy; 2 89686 Endoscopy Unit, Hopital Prive Jean Mermoz, Lyon, France; 3 Endoscopy Unit, Paoli Calmettes Institute, Marseille, France; 4 19051 Gastroenterology and Digestive Endoscopy Unit, University of Verona, Verona, Italy; 5 472771 Gastroenterology and Endoscopy Unit, ASST Rhodense, Garbagnate Milanese, Italy; 6 Medicine, Haukeland University Hospital, Bergen, Norway; 7 472771 Oncology Unit, ASST Rhodense, Garbagnate Milanese, Italy; 8 Endoscopy Unit, Clinique du Trocadéro, Paris, France; 9 18654 Epidemiology and Biostatistics Facility, Fondazione Policlinico Universitario Agostino Gemelli IRCCS, Roma, Italy

**Keywords:** Endoscopic ultrasonography, Pancreas, Intervention EUS, Endoscopy Upper GI Tract, RFA and ablative methods

## Abstract

**Background and study aims:**

Endoscopic ultrasound-guided radiofrequency ablation (EUS-RFA) has been proposed as a minimally invasive alternative to surgery for treatment of both functional (F-) and non-functional (NF-) pancreatic neuroendocrine tumors (PanNETs). We performed a large prospective multicenter study to evaluate safety and effectiveness of EUS-RFA in patients with F- and NF-PanNETs.

**Patients and methods:**

Patients with F- (≤ 2 cm) and NF-PanNETs (15 mm-25 mm) were prospectively enrolled over a 43-month period. The primary aim was safety, defined as rate of adverse events (AEs). The secondary aim was effectiveness defined as complete disappearance of the hormonal secretion syndrome in F- PanNETs, whereas for NF-PanNETs, complete response was defined as absence of enhancing tissue and of detectable lesion at magnetic resonance imaging/computed tomography scan and Gallium-positron emission tomography, respectively. The EUSRA 19G needle was used in all patients. After treatment completion, follow-up was performed for 1 year.

**Results:**

During the study period, a total of 60 patients were enrolled, 30 with insulinomas and 30 with NF-PanNETs (mean lesion size 14.5 ± 4.5 mm). In 78.3% of patients, a single RFA session was performed. Overall, AEs occurred in nine of 60 patients (15%), in four patients (13.3%) with insulinomas and in five (16.7%) with NF-PanNETs, with only one severe AE. Complete insulin hypersecretion symptom resolution was obtained in 29 of 30 patients (96.7%) with insulinomas, whereas for NF-PanNETs, complete radiological response was obtained in 22 of 25 patients (88%) with long-term follow up.

**Conclusions:**

Our prospective international multicenter study demonstrated that EUS-RFA is highly safe and effective for the treatment for both F- and NF-PanNETs.

## Introduction

Functional (F-) and non-functional (NF-) pancreatic neuroendocrine tumors (PanNETs) historically have been treated by surgical resection. Although significant benefit in terms of survival has been observed 1 2 , substantial short- and long-term adverse events (AEs) have been reported. In particular, according to a recent systematic review including 62 studies, occurrence of postoperative pancreatic fistula, delayed gastric emptying, and hemorrhage ranged from 14% to 58%, 5% to 18%, and in 1% to 7% of cases, depending on the surgical approach utilized 3 . Moreover, in-hospital mortality was described in 3% to 6% of patients, whereas long-term pancreatic endocrine and exocrine insufficiencies have been reported to occur in 18% and 33% of cases, respectively 4 5 .

Rate and severity of surgically related AEs have stimulated the search for alternative less invasive loco-regional therapeutic interventions. Endoscopic ultrasound-guided ethanol injection (EUS-EI) directly into the PanNET lesion using standard 22G needles for fine-needle aspiration was the first method introduced in clinical practice 6 . Available studies demonstrated high rates of success in F-PanNETs, whereas in NF-PanNETs, the success rate dropped to 50% to 60% 7 , with a significant rate of AEs. Indeed, technical limitations such as uneven distribution of the injected ethanol inside the target lesion, difficulty in controlling ethanol diffusion to the normal pancreatic lesion-surrounding tissue with a significant risk for acute pancreatitis, and lack of technique standardization (optimal needle size, volume and concentration of ethanol) precluded full dissemination of EUS-EI for treatment of these tumors 8 .

To overcome EUS-EI limitations, a different approach utilizing EUS-guided radiofrequency ablation (EUS-RFA), which permits better control through energy modulation and ablation time and includes a cooling system to improve energy diffusion, has been developed. Various systematic reviews and meta-analyses have been published in which EUS-RFA demonstrated encouraging results for treatment of both non-functioning (NF-) and F-PanNETs 9 10 .

Available data, however, derived mostly from case reports or small case series, whereas prospective data that would avoid selection biases typical of retrospective studies are scanty. To fill this gap, we performed a multicenter prospective international study, with the primary aim to evaluate safety of EUS-RFA for treatment of both F- and NF-PanNETs. Secondary aims were to determine effectiveness of EUS-RFA and to evaluate the rate of patients requiring secondary surgery.

## Patients and methods

### Patients

This international, multicenter, prospective study was approved by the ethics committee (EC) of the Fondazione Policlinico Universitario A. Gemelli IRCCS, Rome, Italy on January 2019 (protocol no. 4171/19). Initially, 10 additional centers agreed to participate in the study. However, with the advent of the SARS-Cov-2 infection, almost all centers dropped out and other additional European centers were invited to participate in the study, for a total of seven enrolling centers. The study was subsequently approved by the local EC of all the other additional six participating centers. The protocol was registered at ClinicalTrials.gov (NCT03834701). All patients provided written informed consent before the procedure. All authors had access to the study data and reviewed and approved the final manuscript.

Consecutive patients with clinical suspicion of insulinomas or histologically proven NF-PNETs were evaluated for study eligibility. Inclusion criteria common for all patients included: ≥ 18 years or < 80 years old; able to provide written informed consent; pancreatic lesion located > 1 mm from the main pancreatic duct (MPD); presence of homogeneous enhancement at contrast harmonic EUS (CH-EUS), single pancreatic lesion on computed tomography (CT), and/or magnetic resonance imaging (MRI), and/or EUS. Specific inclusion criteria for patients with suspected insulinomas were definitive diagnosis of a clinical syndrome related to excessive insulin secretion based on fasting serum glucose, insulin and C-peptide levels) 11 and lesion diameter ≤ 20 mm. Specific inclusion criteria for patients with NF-PanNETs were: lesion diameter 15 to 25 mm, G1 or G2 (Ki67 ≤ 5%) grading on histological examination of EUS-guided biopsy samples obtained with fine-needle biopsy needles, uptake confined to the pancreas without lymph nodes, liver, and other distant metastases at gallium positron emission tomography (PET)-CT, and absence of symptoms or other features suggestive of an aggressive form of NF-PanNETs (presence of calcification, or dilation of the MPD).

Patients with a pancreatic lesion located < 1 mm from the MPD, past medical history of a known bleeding disorder that could not be sufficiently corrected with blood products, on anticoagulants that could not be discontinued or pregnancy were excluded from the study.

### Endpoints and definitions

#### Primary endpoint

The primary endpoint was safety, defined as the percentage of AEs related to EUS-RFA procedures, which were classified according to the American Society for Gastrointestinal Endoscopy (ASGE) 12 lexicon. Based on timing, AEs were separated into peri-procedural (within 24 hours), post-procedural (up to 14 days), and late (any time after 14 days). Short- and long-term AEs were evaluated by telephone contact or outpatient visits on days 7, 15, 30, and 90 after EUS-RFA and every 3 months thereafter for the remaining follow-up period.

#### Secondary endpoint

The secondary endpoint was efficacy of EUS-RFA at 12 months defined for insulinomas as complete disappearance of symptoms related to hyper-hormonal secretion syndrome. Those with symptom recurrence within1e year were considered non-responders after excluding presence of metachronous lesions on imaging (CT, and/or MRI, and/or EUS).

For NF-PanNETs, treatment efficacy was determined by response defined as complete in case where the pancreatic lesion could not be detected or there was no enhancing tissue at the tumor site at on Gallium PET and MRI/CT. Partial response (PR) was identified by persistence of a detectable pancreatic lesion and/or enhancing tissue ≤ 30% of initial tumor volume on gallium PET and MRI/CT, with negative lymph node and distant metastases. Finally, presence of a detectable pancreatic lesion and enhancing tissue > 30% of initial tumor volume on gallium PET and MRI/CT, with or without positive lymph node and distant metastases, was classified as a completely absent response.

### EUS radiofrequency ablation procedure

A kickoff meeting was held in Rome to establish a common procedure protocol. All patients were hospitalized to undergo the procedure for a minimum of 1 or 2 days in accordance with each national reimbursement system. Intrarectal indomethacin or diclofenac 100-mg suppository was administered before the procedure for acute pancreatitis prophylaxis. Antibiotics were given according to the local protocol before the procedure to prevent infection. After informed consent was obtained, the procedure was performed with a therapeutic linear-array echoendoscope (38-J10UT, Pentax Medical, Tokyo, Japan; or GF-UCT180, Olympus Medical Europe, Hamburg, Germany). EUS-RFA was done utilizing a 19G needle with active exposed tip (EUSRA; Taewoong Medical, Gyeonggi-do, South Korea), a dedicated generator (Viva Combo, STARmed), and a cooling system. The exposed tip length and power wattage were standardized and chosen according to tumor size. For lesions < 10 mm in diameter, a 5-mm exposed tip with a 30-watt setting was used, whereas for lesions > 10 mm in diameter, the 10- or 15-mm exposed tips and 50 watts were utilized.

Contrast-enhanced EUS, using an intravenous 4.8-mL SonoVue injection (Bracco International B.V., Amsterdam, Netherlands) through an antecubital vein with a 20G catheter followed by a 10-mL saline solution flush was performed before and after EUS-RFA to evaluate for residual tissue to be ablated. After standard EUS scanning and Doppler examination to exclude intervening vessels, the electrode needle was inserted into the central portion of the lesion under direct EUS guidance. This was decided because RF current diffuses laterally from the needle in both directions and an extensive ablation area can be obtained just by placing the needle in the middle. This decision was also made to standardize RFA treatment.

Radiofrequency current was administered and stopped until a significant increase in impedance, as indicated by the generator, was detected. If necessary, the procedure was repeated by reinserting the needle into different non-ablated portions of the lesion until the largest possible ablation of the tumor was obtained for a maximum of three RFA ablations per treatment session. After the first EUS-RFA session, contrast-enhanced EUS was scheduled 1 month later. In patients with persistent symptoms and residual enhancing tissue, a maximum of three additional sessions of EUS-RFA could be performed as part of the present study criteria.

### Sample size calculation

Based on the number of centers involved and the estimated enrollment rate per center of six to eight patients with PanNETs per year, we hypothesized to enroll at least 30 patients in each group (insulinomas and NF-PanNETs). An average estimate of 60 patients, assuming a power of 80% and a two-sided 95% confidence interval with a significance level of 0.05, is consistent with an estimated moderate effect size, equal to 0.4. Assuming a single-group (one-arm) design to test whether the proportion effect size is different from 0 (H0: h = 0 versus H1: h ≠ 0), with a Type I error rate (α) of 0,05 and an 80% power, 60 subjects would be needed to detect a moderate effect size of 0.4. Sample size was computed using PASS 2019, v19 by applying a two-sided, one-sample Z-test.

### Statistical analysis

Data were collected using an online case record form. Clinical and demographic characteristics of patients were described using descriptive statistics. Qualitative data were expressed as absolute and relative percentage frequencies, whereas quantitative variables were expressed as mean and standard deviation (SD) or median and interquartile range (IQR), as appropriate. The Shapiro–Wilk test was applied to verify Gaussian distribution of the quantitative variables. Between-group differences in each substudy (presence vs. absence of AEs, qualitative tumor variables) was performed using the Chi squared test or Fisher-Freeman-Halton’s exact test, as appropriate. Ordinal qualitative variables and quantitative data were instead compared using either the Student’s *t* -test or the non-parametric Mann–Whitney U test, as appropriate. Statistical significance was set at *P* < 0.05. The whole analysis was performed with R software v4.2.2 (CRAN, R Core 2022, Wien, Austria).

## Results

### Study population

Between April 2019 and November 2022, a total of 65 patients were evaluated at seven tertiary referral centers. Two patients with insulinomas and three with NF-PanNETs were excluded because the lesion directly involved the MPD, which appeared dilated by compression from the lesion. There were no exclusions related to presence of multiple lesions nor observation of heterogeneous enhancement at CH-EUS. Overall, 60 patients, 30 with insulinomas and 30 with NF-PanNETs were enrolled in the study. None of the enrolled patients were included in previous published studies. Table 1 shows demographic, clinical, and procedure characteristics of enrolled patients. Overall, mean age was 65.92 ± 16.0 years, without sex predominance. Mean lesion diameter was 14.5 ± 4.5 mm. The majority of patients underwent a single RFA session (78.3%). Fifty-five patients completed the 12-month follow up, whereas two patients with NF-PanNETs were lost after a median of 10.5 months and other three patients with NF-PanNETs did not perform scheduled FU imaging to assess efficacy.

**Table:** **Table 1** General characteristics of the study sample.

	Overall (N = 60)	Insulinomas (N = 30)	NF-PanNETs (N = 30)
Age, years	65.92 (16.0)	61.9 (16.5)	70.0 (14.7)
Gender			
Male	30 (50%)	10 (33.3)	20 (66.7)
Female	30 (50%)	20 (66.7)	10 (33.3)
Tumor site			
Body	14 (23.3%)	5 (16.7)	9 (30)
Head/uncinate	21 (35.0%)	13 (43.3)	8 (26.7)
Neck	10 (16.7%)	6 (20)	4 (13.3)
Tail	15 (25.0%)	6 (20)	9 (30)
Tumor maximum diameter, mm	14.5 (4.5)	11.9 (3.4)	17.1 (3.9)
Lesion distance from MPD, mm	3 (1.6)	2.5 (1.0)	3.5 (1.9)
Number of RFA sessions			
1	47 (78.3%)	25 (83.3)	22 (73.3)
≥ 2	13 (21.7%)	5 (16.7)	8 (27.7)
MPD, main pancreatic duct; NF-PanNET, non-functional pancreatic neuroendocrine neoplasm; RFA, radiofrequency ablation. *Quantitative data are expressed as mean and standard deviation (SD), whereas qualitative as absolute and relative percentage frequencies.			

### Primary outcome

Overall, AEs were observed in nine of 60 patients (15%), in particular in four patients (13.3%) with insulinomas and in five (16.7%) with NF-PanNETs ( Table 1 ). Eight AEs occurred peri-procedurally, including five cases of mild acute pancreatitis all managed conservatively without prolongation of hospital stay or occurrence of any sequelae. Additional peri-procedural AEs included development of a peripancreatic hematoma in a patient with pancreatic head insulinoma, which did not require blood transfusion or prolongation of hospitalization, and intragastric bleeding in another patient with a 22-mm, G1, NF-PanNET of the pancreatic body, which required blood transfusion and prolongation of hospital stay for four additional days.

Finally, two cases of injury of the MPD occurred. The first patient with a 10-mm insulinoma in the pancreatic neck, distant 4 mm from the MPD, developed pain 7 months after a single EUS-RFA session. Pancreatic duct stenosis was demonstrated on MRI and managed endoscopically with pancreatic stent insertion followed by removal at 3 months, with complete symptom resolution. In another patient with an 18-mm, G1, pancreatic head NF-PanNET distant 5 mm from the MPD, peritoneal effusion of pancreatic juice secondary to MPD damage occurred within 48 hours after the second and final session of EUS-RFA. The patient was managed endoscopically with pancreatic sphincterotomy and pancreatic stent insertion and surgically with laparoscopic washing and drainage of the abdominal cavity. The patient was discharged 4 days after the RFA procedure without further sequelae. This was the only severe AE that occurred in our study population.

We further assessed potential factors associated with occurrence of EUS-RFA-related AEs ( Table 2 ). Overall, no demographic variables, lesion site and size, distance from the MPD, PanNET type (insulinoma vs. NF-), or number of EUS-RFA sessions performed (one vs. more) were associated with development of AEs.

**Table:** **Table 2** Differences between patients with and without complications (n = 60).

	Complications		*P* value
	Yes (n = 9)	No (n = 51)	
Age, years	69.4 (17.1)	65.3 (16)	0.478
Gender			1.000
Male	4 (44.4%)	26 (51%)	
Female	5 (55.6%)	25 (49%)	
Tumor site			1.000
Head+uncinate	3 (33.3%)	18 (35.9%)	
Body+tail+neck	6 (66.7%)	33 (64.71%)	
Tumor maximum diameter, mm	15.0 (4.4)	14.4 (4.7)	0.728
Lesion distance from MPD, mm	3.1 (1.1)	3.0 (1.6)	0.824
PanNET type			1.000
Insulinoma	4 (44.4%)	26 (51%)	
NF-PanNET	5 (55.6%)	25 (49%)	
Number of RFA sessions			1.000
1	7 (77.8%)	41 (80.4%)	
≥ 2	2 (22.2%)	10 (19.6%)	
MPD, main pancreatic duct; NF, non-functional; PanNET, pancreatic neuroendocrine neoplasm; RFA, radiofrequency ablation. *Quantitative data are expressed as mean and standard deviation (SD) and compared by Student’s *t* -test, whereas qualitative as absolute and relative percentage frequencies and assessed by Fisher-Freeman-Halton’s test.			

### Secondary outcome

In patients with insulinomas, symptoms related to insulin hypersecretion completely resolved at 12 months in all but one of 30 enrolled patients (96.7%). Five patients, due to persistent symptoms and evidence of residual vital tissue, underwent two sessions of EUS-RFA obtaining complete symptom resolution and lesion ablation without enhancing tissue at CH-EUS. One patient with a 17-mm pancreatic head insulinoma underwent three EUS-RFA sessions because of persistent symptoms and enhancing tissue on CH-EUS. Three months after the third EUS-RFA session, the patient experienced symptom recurrence for which she underwent a fourth EUS-RFA session with symptom resolution. In this patient, however, based on the study endpoint definition, EUS-RFA treatment was considered a failure.

Efficacy of EUS-RFA in NF-PanNETs was evaluated in 25 patients because in the other five cases, scheduled imaging was not performed. Two patients were lost at follow up at a median of 10.5 months before performing scheduled imaging studies. In the other two, worsening of preexisting clinical conditions (heart failure and ischemic stroke) precluded subsequent follow-up examinations, whereas the remaining one refused to undergo any further imaging studies. At 12 months from the last EUS-RFA session, complete response was obtained in 22 of 25 patients (88%), whereas in the other three (12%), a PR was observed.

Subsequent management of patients with PR was discussed during a multidisciplinary meeting, in which the decision also took into consideration patient preference. One patient underwent surgery for persistent enhancing tissue of a 25-mm pancreatic tail NF-PanNET. A second patient with an 18-mm pancreatic head NF-PanNET was scheduled for a further session of EUS-RFA, which was performed without occurrence of intra-procedural AEs and without evidence of enhancing tissue at CH-EUS at 1 month. The third patient, with a 17-mm, G2, NF-PanNET of the uncinate process, refused subsequent interventions and radiological follow up was performed at 12 months without evidence of disease progression from the initial PR.

## Discussion

We performed a multicenter European prospective study with the primary aim of evaluating safety of EUS-RFA treatment in a large cohort of consecutive patients with both F- and incidentally discovered small NF-PanNETs. Overall, AEs occurred in 15% of patients, with no differences between those with F- (all insulinomas, 13.3%) or NF-PanNETs (16.7%), and with only a single severe AE.

The goal of EUS-RFA treatment of PanNETs differs in functional and NF- tumors. In F-PanNETs, ablation of enough tissue to obtain cessation of the clinical hormonal syndrome is required, without need to treat the entire lesion because of the low malignant potential 10 . Conversely, in NF-PanNETs, all of the lesion needs to be completely ablated, without leaving remnant vital tissue including tumoral margins. This makes EUS-RFA treatment in patients with NF-PanNETs more complex with theoretically increased risk for AEs.

This crucial therapeutic aspect may influence AE and effectiveness rates depending on the type of treated PanNETs. In our study, AEs occurred at similar rates in both F- and NF-PanNETs, similar to results of a very recent systematic review and meta-analysis 13 . It is possible that the smaller diameter of F-PanNETs as compared with NF-PanNETs could have increased risk of AEs, as observed in a retrospective study by Marx et al. in which EUS-RFA-induced AP occurred in 14.8% of patients, all with lesions ≤ 10 mm 14 . Importantly in the systematic review and meta-analysis reported above, severe AEs were rare (0.7%) (13), as in our study (1.7%). In our patient with a severe AE, damage of MPD with leak of pancreatic juice in the peritoneum occurred and required performance of endoscopic retrograde pancreatography with pancreatic sphincterotomy and surgical washing and drainage of the abdominal cavity. The patient had a lesion 5 mm away from the MPD. Of interest, another patient with a lesion located 4 mm from the MPD developed pancreatic ductal stenosis. Thus, our caution in including only patients with a distance of the lesion > 1 mm from MPD failed to prevent this AE. Reasons for this behavior remain unclear and are in contrast with the conclusions of a large multicenter retrospective French study on EUS-RFA treatment of not only NETs but also pancreatic metastases and intraductal papillary mucinous cysts 15 . The authors found that distance from the MPD < 1 mm was the only predictive factor for occurrence of AEs, in both univariate and multivariate analyses. Prophylactic MPD stenting has been proposed 16 , but its value remains to be established.

In line with data from two published systematic reviews and meta-analyses 13 , in the present study treatment efficacy was very high in both insulinomas (96.7%) and NF-PanNETs (88%). Regarding insulinomas, a retrospective propensity-matched analysis comparing EUS-RFA (89 patients) with surgical resection (89 patients) reported clinical efficacy to be similar between the two treatment groups (95.5% versus 100%, *P* = 0.160). Conversely, overall (18% vs 61.8%, *P* < 0.001) and severe (0% versus 15.7%, *P* < 0.001) AEs occurred more frequently in surgical patients and caused a consequent significantly longer hospital stay than EUS-RFA (11.1 ± 9.7 days vs. 3.4 ± 3.0 days, *P* < 0.001). The results of this study and our prospective data, despite contrasting with the conclusions of recent ENETS guidelines 17 , strongly suggests that EUS-RFA can become the standard of care for the large majority of patients with F-Pan-NETs. A multicenter randomized controlled trial to directly compare these two treatment modalities in patients with insulinomas is ongoing 18 .

Clinical effectiveness of EUS-RFA in our patients with small, resectable NF-PanNETs was 88%, which is similar to results of recent meta-analyses 13 19 . These very encouraging data deserve a deep discussion. First of all, the most important issue is which patients should be treated with EUS-RFA. Recent updated guidelines from the ENETS suggest surveillance for patients with lesions < 2 cm 20 . In real life, however, in two retrospective surgical series, about 28% to 29% of patients with NF-Pan-NETs < 2 cm underwent surgical resection, which caused grade III AEs in 24% and 18% of the patients, respectively 5 21 . In a more recent prospective multicenter study, 19% of patients with small NF-PanNETs underwent resection at baseline, with grade 1 to 4 AEs in 32% of them 22 .

In terms of lesion size, we included lesions between 15 and 25 mm, whereas in another ongoing study, the RFANET study (NCT04520932), the cut-off was between 10 and 20 mm. Because of the high rate of AEs in patients with lesions around 10 mm 23 and the reported association with a positive response to EUS-RFA of PanNETs with a size at EUS ≤ 18 mm 19 , it is our opinion that only lesions between 14 to 15 mm to 20 mm should be considered for EUS-RFA treatment. In patients with a growing lesion, EUS-RFA, when indicated, should be applied when the tumor has not become too big to increase the chance of obtaining a complete response.

Should EUS-RFA be offered to patients only at high surgical risk? Major skepticism expressed by oncologists and surgeons about EUS-RFA treatment of NF-PanNETs is the impossibility of verifying achievement of R0 resection margins and the uncertainty about long-term outcomes. The only available study with more than 3 years of follow up is limited to 12 patients 24 and reported persistence of complete response in all but one case (91.6%) after a mean follow-up of 45.6 months. The patient with recurrent disease underwent EUS-FNB that detected a G1 NF-PanNET and was offered a repeat EUS-RFA treatment but refused. A step-up approach has been proposed in which EUS-RFA for NF-PanNETs is utilized first because of the reduced rate of AEs, leaving surgery as a backup for cases of incomplete response or with recurrent disease 7 . Based on this approach, all patients with lesions ≤ 2 cm, Ki-67 ≤5, without symptoms or MPD dilatation might be considered for EUS-RFA as a first-line treatment, with surgery reserved for those who do not respond or relapse. Future studies on large cohorts of patients prospectively enrolled are needed to define exact indications for EUS-RFA in patients with NF-PanNETs.

The present study has some limitations, including a relatively short follow up and the use of symptoms resolution to define efficacy of RFA treatment in F-PanNETs. Strengths include its prospective and multicenter nature, with the largest number of patients prospectively treated so far, with a standardized EUS-RFA procedure.

## Conclusions

In conclusion, EUS-RFA for PanNETs is safe and associated with an extremely low rate of severe AEs. The high treatment successful rate in F-PanNET will most likely soon transform EUS-RFA as a standard of care for the large majority of these patients. More data, on a large cohort of NF-PanNETs patients with a long follow up, are needed before this approach can be adopted on a widespread basis.

## References

[1] Falconi M Eriksson B Kaltsas G ENETS Consensus Guidelines Update for the Management of Patients with Functional Pancreatic Neuroendocrine Tumors and Non-Functional Pancreatic Neuroendocrine Tumors Neuroendocrinology 2016 103 153 171 26742109 10.1159/000443171 PMC4849884

[2] Hill JS McPhee JT McDade TP Pancreatic neuroendocrine tumors: the impact of surgical resection on survival Cancer 2009 115 741 751 10.1002/cncr.24065 19130464

[3] Jilesen AP van Eijck CH in't Hof KH Postoperative complications, in-hospital mortality and 5-year survival after surgical resection for patients with a pancreatic neuroendocrine tumor: A systematic review World J Surg 2016 40 729 748 26661846 10.1007/s00268-015-3328-6 PMC4746219

[4] Falconi M Mantovani W Crippa S Pancreatic insufficiency after different resections for benign tumours Br J Surg 2008 95 85 91 10.1002/bjs.5652 18041022

[5] Partelli S Mazza M Andreasi V Management of small asymptomatic nonfunctioning pancreatic neuroendocrine tumors: Limitations to apply guidelines into real life Surgery 2019 166 157 163 31109657 10.1016/j.surg.2019.04.003

[6] Jurgensen C Schuppan D Neser F EUS-guided alcohol ablation of an insulinoma Gastrointest Endosc 2006 63 1059 1062 10.1016/j.gie.2005.10.034 16733126

[7] Rimbas M Rizzatti G Tosoni A Small nonfunctional pancreatic neuroendocrine neoplasms: Time for a step-up treatment approach? Endosc Ultrasound 2023 12 1 7 10.4103/EUS-D-22-00028 36510866 PMC10134916

[8] Lakhtakia S Therapy of pancreatic neuroendocrine tumors: Fine needle intervention including ethanol and radiofrequency ablation Clin Endosc 2017 50 546 551 10.5946/ce.2017.167 29207860 PMC5719904

[9] Larghi A Rizzatti G Rimbas M EUS-guided radiofrequency ablation as an alternative to surgery for pancreatic neuroendocrine neoplasms: Who should we treat? Endosc Ultrasound 2019 8 220 226 31249164 10.4103/eus.eus_28_19 PMC6714479

[10] Di Gialleonardo L Tripodi G Rizzatti G Endoscopic ultrasound-guided locoregional treatments for solid pancreatic neoplasms Cancers (Basel) 2023 15 4718 10.3390/cancers15194718 37835413 PMC10571848

[11] Cryer PE Axelrod L Grossman AB Evaluation and management of adult hypoglycemic disorders: an Endocrine Society Clinical Practice Guideline J Clin Endocrinol Metab 2009 94 709 728 10.1210/jc.2008-1410 19088155

[12] Cotton PB Eisen GM Aabakken L A lexicon for endoscopic adverse events: report of an ASGE workshop Gastrointest Endosc 2010 71 446 454 10.1016/j.gie.2009.10.027 20189503

[13] Armellini E Facciorusso A Crino SF Efficacy and safety of endoscopic ultrasound-guided radiofrequency ablation for pancreatic neuroendocrine tumors: A systematic review and metanalysis Medicina (Kaunas) 2023 59 359 10.3390/medicina59020359 36837560 PMC9963038

[14] Marx M Godat S Caillol F Management of non-functional pancreatic neuroendocrine tumors by endoscopic ultrasound-guided radiofrequency ablation: Retrospective study in two tertiary centers Dig Endosc 2022 34 1207 1213 10.1111/den.14224 34963025

[15] Napoleon B Lisotti A Caillol F Risk factors for EUS-guided radiofrequency ablation adverse events in patients with pancreatic neoplasms: a large national French study (RAFPAN study) Gastrointest Endosc 2023 98 392 9 e1 37059368 10.1016/j.gie.2023.04.003

[16] Choi JH Seo DW Song TJ Endoscopic ultrasound-guided radiofrequency ablation for management of benign solid pancreatic tumors Endoscopy 2018 50 1099 1104 10.1055/a-0583-8387 29727904

[17] Hofland J Falconi M Christ E European Neuroendocrine Tumor Society 2023 guidance paper for functioning pancreatic neuroendocrine tumour syndromes J Neuroendocrinol 2023 35 e13318 10.1111/jne.13318 37578384

[18] Crino SF Partelli S Napoleon B Study protocol for a multicenter randomized controlled trial to compare radiofrequency ablation with surgical resection for treatment of pancreatic insulinoma Dig Liver Dis 2023 55 1187 1193 37407318 10.1016/j.dld.2023.06.021

[19] Imperatore N de Nucci G Mandelli Ed Endoscopic ultrasound-guided radiofrequency ablation of pancreatic neuroendocrine tumors: a systematic review of the literature Endosc Int Open 2020 8 E1759 E1764 10.1055/a-1261-9605 33269308 PMC7671767

[20] Kos-Kudla B Castano JP Denecke T European Neuroendocrine Tumour Society (ENETS) 2023 guidance paper for nonfunctioning pancreatic neuroendocrine tumours J Neuroendocrinol 2023 35 e13343 10.1111/jne.13343 37877341

[21] Mintziras I Keck T Werner J Indications for resection and perioperative outcomes of surgery for pancreatic neuroendocrine neoplasms in Germany: an analysis of the prospective DGAV StuDoQ|Pancreas registry Surg Today 2019 49 1013 1021 10.1007/s00595-019-01838-1 31240463

[22] Partelli S Massironi S Zerbi A Management of asymptomatic sporadic non-functioning pancreatic neuroendocrine neoplasms no larger than 2 cm: interim analysis of prospective ASPEN trial Br J Surg 2022 109 1186 1190 35986682 10.1093/bjs/znac267 PMC10364756

[23] Marx M Trosic-Ivanisevic T Caillol F EUS-guided radiofrequency ablation for pancreatic insulinoma: experience in 2 tertiary centers Gastrointest Endosc 2022 95 1 1256 1263 10.1016/j.gie.2021.11.045 34902374

[24] Barthet M Giovannini M Gasmi M Long-term outcome after EUS-guided radiofrequency ablation: Prospective results in pancreatic neuroendocrine tumors and pancreatic cystic neoplasms Endosc Int Open 2021 9 E1178 E1185 10.1055/a-1479-2199 34447860 PMC8383082

